# Posterior dislocation of elbow with brachial artery injury

**DOI:** 10.4103/0974-2700.66560

**Published:** 2010

**Authors:** Karun Jain, Y Shashi Kumar, Mruthyunjaya, R Ravishankar, Ayyappan V Nair

**Affiliations:** Department of Orthopaedics, JSS Medical College and Hospital, Mysore - 570 004, India

Sir,

Although isolated closed posterior elbow dislocation occurs frequently, associated brachial artery injury is an uncommon complication, which is rarely encountered.[1] Complications, such as intermittent claudication or gangrene of the hand are possible if brachial artery flow is not restored.

**Figure 1:**
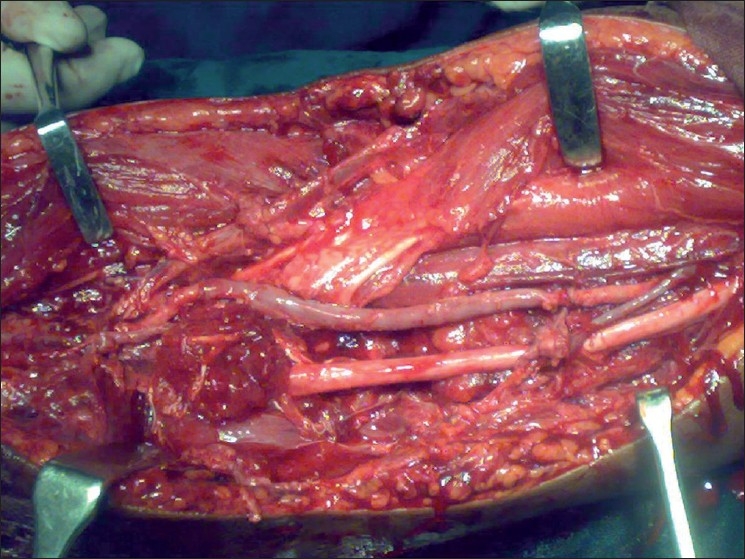
Interposition reversed sephanous vein graft to bridge the 12 cm gap between the lacerated ends of brachial artery

A 16-year-old boy presented to the emergency department with pain, swelling, and deformity of the right elbow following a fall from a tree, on outstretched hand. There was no history of head injury or loss of consciousness or bleeding from any orifices or blunt abdominal injury. X-rays showed a posterior dislocation of elbow. There was no radial pulses palpable hence patient was taken up for emergency surgery. The patient had lacerated his radial artery which was repaired using a “reversed saphenous vein interposition graft [Figure 1].

The patient made a good post operative recovery and the function of the hand was preserved.

The pearl is that emergency exploration and repair or reconstruction of the arterial injuries provide good functional outcome and help us to save “the working hand.” We draw attention to the need of meticulous clinical examination of all cases with doubtful vascularity, as Doppler study and Angiogram are not easily accessible in many casualty setups, for surgical decision-making.

To conclude, until this date only a handful of cases have been reported, where a patient with closed elbow dislocation with or without associated fractures.[2] Our case being one such, points to the necessity for not to overlook such a complication, though rare!
